# CDCA8 induced by NF-YA promotes hepatocellular carcinoma progression by regulating the MEK/ERK pathway

**DOI:** 10.1186/s40164-022-00366-y

**Published:** 2023-01-13

**Authors:** Erbao Chen, Yu He, Jing Jiang, Jing Yi, Zhilin Zou, Qiuzi Song, Qingqi Ren, Zewei Lin, Yi Lu, Jikui Liu, Jian Zhang

**Affiliations:** 1 Department of Hepatobiliary and Pancreatic Surgery, Peking University Shenzhen Hospital, Shenzhen, 518036 Guangdong China; 2 School of Medicine, Southern University of Science and Technology, Shenzhen, 518055 Guangdong China; 3 Department of Pathology, Peking University Shenzhen Hospital, Shenzhen, Guangdong China; 4 Department of Ophthalmology, Affiliated Eye Hospital of Wenzhou Medical University, Wenzhou, Zhejiang China; 5 Guangdong Provincial Key Laboratory of Cell Microenvironment and Disease Research, Shenzhen, Guangdong China

**Keywords:** Hepatocellular carcinoma, Cell division cycle associated 8, NF-YA, Cancer metastasis, Prognosis

## Abstract

**Background:**

Hepatocellular carcinoma (HCC) is one of the most lethal malignant tumors. Cell division cycle associated 8 (CDCA8) is an important multifactorial regulator in cancers. However, its up and downstream targets and effects in HCC are still unclear.

**Methods:**

A comprehensive bioinformatics analysis was performed using The Cancer Genome Atlas dataset (TCGA) to explore novel core oncogenes. We quantified CDCA8 levels in HCC tumors using qRT-PCR. HCC cell’s proliferative, migratory, and invasive abilities were detected using a Cell Counting Kit-8 (CCK-8) assay, 5-ethynyl-2′-deoxyuridine (EdU) assay, clone formation, and a Transwell assay. An orthotopic tumor model and tail vein model were constructed to determine the effects of CDCA8 inhibition in vivo. The mechanism underlying CDCA8 was investigated using RNA sequencing. The prognostic value of CDCA8 was assessed with immunohistochemical staining of the tissue microarrays.

**Results:**

CDCA8 was identified as a novel oncogene during HCC development. The high expression of CDCA8 was an independent predictor for worse HCC outcomes both in publicly available datasets and in our cohort. We found that CDCA8 knockdown inhibited HCC cell proliferation, colony formation, and migration by suppressing the MEK/ERK pathway in vitro. Moreover, CDCA8 deficiency significantly inhibited tumorigenesis and metastasis. Next-generation sequencing and laboratory validation showed that CDCA8 silencing inhibited the expression of TPM3, NECAP2, and USP13. Furthermore, NA-YA overexpression upregulated the expression of CDCA8. CDCA8 knockdown could attenuate NF-YA-mediated cell invasion in vitro. The expression of NF-YA alone or in combined with CDCA8 were validated as significant independent risk factors for patient survival.

**Conclusion:**

Our findings revealed that the expression of CDCA8 alone or in combined with NF-YA contributed to cancer progression, and could serve as novel potential therapeutic targets for HCC patients.

**Supplementary Information:**

The online version contains supplementary material available at 10.1186/s40164-022-00366-y.

## Induction

Hepatocellular carcinoma (HCC), which accounts for about 85–90% of liver malignancies, ranks as the sixth most-common malignant neoplasm and the third-highest cause of cancer-associated mortality worldwide [1]. Unfortunately, patients are often diagnosed with HCC at advanced stages, so treatment efficacy is limited and poor. Nowadays, although many strategies, including immunotherapy and targeted drug have been employed to treat HCC, up to 70% of HCC patients will relapse within 5 years after radical hepatectomy [2]. Thus, identifying other novel targets and exploring the underlying mechanism of HCC development is especially important for improving HCC prognosis.

The abnormal expression of cyclins and cyclins associated-proteins is a hallmark of cancer development [3]. Cell division cycle associated 8 (CDCA8) is a necessary component of chromosomal passenger complex (CPC), formed by CDCA8, survivin, INCENP, and Aurora B. CDCA8 is essential for chromosomal segregation during mitosis. Aberrantly expressed CDCA8 leads to polyploidy, mitotic failure, and abnormal cell division, suggesting it plays an important role in cellular homeostasis [4]. Accumulating evidence suggests that CDCA8 dysregulation is observed in many cancers and is essential for cell survival and metastasis for gastric [5], kidney, colorectal [6], lung [7], and breast cancers [8]. As for the mechanism of CDCA8 overexpression, UAP1L1 is identified as a critical factor for CDCA8 expression, and promotes cell growth, migration, invasion, and apoptosis in prostate cancer [9]. KIF18 was found to bind to the promoter region of CDCA8 and enhance CDCA8 expression, as well as increase cell proliferation capacity in pancreatic cancer [10]. A recent bioinformatic analysis showed that CDCA8 was strongly associated with HCC development, occurrence, and metastasis [11–13]. It is reported that nuclear transcription factor Y (NF-Y) can transcriptionally activate the expression of CDCA8 in cancer cell [14]. Unlike the other transcription factors, NF-Y is a heterotrimeric complex consisting of three distinct subunits NF-YA, NF-YB, and NF-YC. Recent study demonstrated that only NF-YA, but not HFD subunits, is negatively associated with the prognosis of HCC [15]. However, previous studies have not verified the correlation between CDCA8 and NF-Y in clinical tissues.

Here, we explored and identified novel up- and down-stream targets of CDCA8 in HCC progression. First, we used transcriptional data from The Cancer Genome Atlas (TCGA) to explore the novel dysregulation of genes with prognostic values, which were validated by our cohort. Second, we observed changes related to HCC phenotypes in vitro and in vivo after enhancing or inhibiting CDCA8. We carried out RNA sequencing to investigate the molecular mechanism underlying CDCA8 silencing in two HCC cell lines. We also observed a significant positive correlation between CDCA8 and NF-YA expression in HCC samples. Further, the expression of CDCA8 alone or in combined with NF-YA were identified as strong independent factors for patients with HCC. In summary, we found that Inhibition of the NF-YA/CDCA8 axis holds promise in treating HCC.

## Methods and materials

### Bioinformatic analysis

GEPIA (http://gepia.cancer-pku.cn/), which sources data from The Cancer Genome Atlas (TCGA, https://tcga-data.nci.nih.gov/tcga/) and the Genotype-Tissue Expression project (GTEx, https://www.gtexportal.org/home/index.html) [16] were used to obtain the differentially expressed genes and prognosis associated-genes. High-throughput RNA sequencing data from multiple cancers was extracted from the Oncomine website (http://www.oncomine.com) [17]. The CDCA8 prognostic curves were obtained from the Kaplan–Meier Plotter (http://kmplot.com/analysis/) [18].

### Cell lines and culture

HepG2, Huh7, PLC/PRF/5 and Bel-7402 cell lines were purchased from the Guangzhou Cellcook Biotech Co.,Ltd (Guangdong, China), HepG2, Huh7, PLC/PRF/5 cell lines were maintained in DMEM medium (Gibco, USA) and the Bel-7402 cell line was maintained in RPMI-1640 medium (Gibco, USA). All the mediums were supplemented with 10% fetal calf serum and 1% Penicillin/Streptomycin at 37 °C in incubators containing 5% CO_2_. Mycoplasma testing of all cells was regularly completed using a Mycoplasma Detection kit (Solarbio, Beijing, China). PD98059 (Sigma-Aldrich, St. Louis, MO, USA) and SCH772984 (MCE, Monmouth Junction, NJ, USA) were used as inhibitors of the MEK/ERK signaling pathway.

### Patient samples and tissue microarray

A total of 144 HCC cancer tissues and corresponding adjacent non-tumor samples were collected from patients who received curative hepatectomies between January 2015 and December 2018 at Peking University Shenzhen Hospital, and were constructed into tissue microarrays (TMAs, named as PKUSZ). Overall survival was defined as the time period between the date of the operation and death or the end of follow-up period (December 2021). Disease-free survival was counted from the date of surgery to the date of recurrence or last follow-up. Another cohort of 71 fresh HCC sample pairs was obtained from HCC patients undergoing curative resection at the Department of Hepatobiliary and Pancreatic Surgery. All cases were reviewed independently by two pathologists. This project was completed in accordance with the Declaration of Helsinki. The use of specimens, clinical characteristics, and follow-up information for research purposes was approved by the Peking University Shenzhen Hospital ethics committee. Informed consent was obtained from all patients before their operations.

### ShRNA or SiRNA transfection, plasmid transfection, and lentiviral transfection

We designed shRNA or siRNA sequences to construct the shCDCA8 or siNF-YA for CDCA8 and NF-YA knockdowns. We cloned CDCA8 shRNA into lentiviral expression GV112 plasmids and established it as a shCDCA8 lentivirus. Cells were transfected with lentivirus using polybrene (5 μg/ml) and then selected with puromycin (3 μg/ml) for 1 week to construct CDCA8 knockdown, or CDCA8 forced expressed cell lines. The shRNA for CDCA8 was purchase from Obio, siRNA for NF-YA knockdowns was purchased from General Biol, and transfected into cells using Lipofectamine 3000. Small interfering RNA of TPM3, NECAP2 and USP13 were purchased from RiboBio (Guangzhou, China). Sequencing for each gene was performed and showed the following: The shRNA sequence for CDCA8 was: cgCCTCCTTTCTGAAAGACTT; The siRNA-1 sequence for NF-YA was: Forward, 5′-GGAGGCCAGCUAAUCACA UTT-3′; Reverse, 5′-AUGUGAUUAGCUGGCCUCCTT-3′; The siRNA-2 sequence for NF-YA was: Forward, 5′-CCUGGUGGACAAGGUCAAATT-3′; Reverse, 5′-UUUGACCUUGUCCACCAGGTT-3′; The siRNA-3 sequence for NF-YA was: Forward, 5′-CCAAACAAUACCACCGUAUTT-3′; Reverse, 5′-AUACGGUGGUAU UGUUUGGTT-3′; The siRNA for NF-YA negative control was: Forward, 5′-UUCUCCGAACGUGUCACGUTT-3′; Reverse, 5′-ACGUGACACGUUCGGAGAA TT-3′.

### Cell counting kit-8, EdU-594 staining assay and colony formation assay

Cell suspensions were placed in 96-well plates (3 × 10^3^ cells per well) and cell proliferation activity was assessed for 24, 48, and 72 h. Subsequently, 10 μl of CCK-8 reagent (Dojindo, Japan) was seeded into each well and cultured in a cell incubator. After 2 h, the absorbance at 450 nm was detected and recorded by a microplate reader.

The cells were seeded into 6-well dishes and incubated with 5-ethynyl-2′-deoxyuridine (EdU, 10 μM) for 2 h. Afterwards, the cells were fixed in 4% paraformaldehyde and permeabilized with 0.3% Triton X-100 for 10 min. Subsequently, the cells stained with DAPI for 5 min at room temperature before detection by fluorescence microscopy.

A total of 1 × 10^3^ cells were plated in 6-well plates and cultured for 14 days. The medium was changed every 3 days. Next, the plates were washed with PBS three times, fixed with 4% paraformaldehyde, and stained with 1% crystal violet. The colony numbers were countered and recorded.

### qRT-PCR and Western blot analysis

Total RNA was isolated from cells using 0.5 mL Trizol, and reverse transcription quantitative PCR was performed using the Prime-Script RT Master Mix (Vazyme, China). qRT-PCR was performed using SYBR Green Realtime PCR Master Mix (Vazyme, China). Samples from each experiment were independently repeated three times. The sequences were as follows: CDCA8: Forward, TTGACTACTTCGCCCTTG, Reverse, CTTCTTCTTCCTCTTCCACTA; TPM3: Forward, 5′-GCACATTGCA GAAGAGGCAG-3′; Reverse, 5′-TCTGTGCGTTCCAAGTCTCC-3′; USP13: Forward, 5′-GCCAAGCACTTAGCGCATTT-3′; Reverse, 5′-CACTTCCCACTCA CTGACCC-3′; NECAP2: Forward, 5′-AACAAGCCCAGAACCCAGAC-3′, Reverse, 5′-CCAGCTGCTCCTTCCTTCTT-3’; SPRYD4: Forward, 5′-CAAGCTGGGGAAC AGCCATA-3′, Reverse, 5′-GAATTTCTGCCCCTTCACGC-3′.

The primary antibodies used in this study were as follows: anti-CDCA8 (Santa Cruz, sc-376635), anti-NF-YA (Santa Cruz, sc-17753), anti-MEK1/2 (CST, 4694), anti-Phospho-MEK1/2 (CST, 9154), anti-p44/42 (CST, 4695), anti-Phospho-p44/42 (CST, 4377), anti-p38 (CST, 8690), anti-rabbit IgG (CST, 4413), and anti-mouse IgG (CST, 7076).

### Cell migration and invasion assays

Transwell chambers with and without Matrigel (BD, America) coating were used to perform migration and invasion assays. A total of 1 × 10^4^ cells were added to the upper chambers; and 800 μl of medium containing 10% FBS was added to the lower chamber. After 48 h incubation, the cells on the lower surface of the membrane were fixed in 4% paraformaldehyde for 20 min, and stained with 0.1% crystal violet for 15 min. Next, 3 randomly selected microscopic fields per well were quantified with a light microscope. The number of migratory or invasive cells was counted.

### RNA-sequencing and analysis

Total RNA isolated from Bel-7402 and HepG2 cells transfected with shCtrl and shCDCA8 lentivirus was subjected to paired-end RNA-seq using an Illumina HiSeq 2000 system based on the manufacturer’s instructions. Read mapping and data analysis were completed for genes that were differentially expressed between the two groups (Fold Change > 2, padj < 0.05).

### In vivo tumor growth and pulmonary metastasis experiment

Five-week-old male Balb/c nude mice were purchased from GemPharmatech (Guangdong, China). 1 × 10^7^ cells transfected with Bel-7402-shCDCA8 or shCtrl were injected into mice flanks to form tumors. After two weeks, the mice were sacrificed, and tumors were obtained. The tumors were next trimmed into 1 mm^3^ square pieces and transplanted into the livers of new mice (n = 6, per group; one control nude mouse died after surgery). After four weeks, we sacrificed the mice, and calculated the volume and tumor weight of tumor tissue. To establish a model of pulmonary metastasis, 2 × 10^6^ cells transfected with shCDCA8, or shCtrl in 150 μl phosphate buffered saline, was injected into mice (n = 6, per group) through their tail veins. After four weeks, the mice were euthanized, and the number of metastatic nodules was counted. The animal research protocol was approved by the Animal Care Committee of Peking University Shenzhen Hospital.

### Chromatin immunoprecipitation (ChIP) assay

4 × 10^6^ cell were lysed in 400 µl SDS Lysis Buffer and then 800 µl cells lysate was sonicated on ice and the fragmented. DNA was visualized on an agarose gel. Anti‑NF-YA or IgG antibodies were added to interact with the target protein‑DNA complex. For chromatin isolation, the sample was centrifuged again at 15,000×*g* for 10 min at 4 °C to remove insoluble material and ChIP dilution buffer (Abs50034, Absin Biotechnology) was added to the collected supernatant. The sample was pre‑cleared with protein A/G‑agarose beads at 4 °C for 2 h with mixing.

### Immunohistochemical staining of the TMA

The process of immunohistochemical staining was performed as in our previous study [19]. Briefly, TMA slides were dewaxed using xylene 10 min and graded ethanol. Then, antigen retrieval was performed using citric acid buffer (pH = 6.0) for 20 min. The sections were treated in 3% H_2_O_2_ for 5 min to eliminate endogenous peroxidase activity. The sections were subsequently incubated with rabbit anti-CDCA8 polyclonal antibodies (1:100 dilution; Santa Cruz, sc-376635) or NF-YA antibody (1:100 dilution; Santa Cruz, sc-17753) overnight at 4 °C and then treated with secondary antibodies for 1 h at 37 °C. Then, these sections were stained with DAB for 5 min in the dark, and 100% hematoxylin was introduced to react for 2 min. Finally, slides were dehydrated with graded alcohol, sealed with neutral balsam, and visualized and scanned with CaseViewer2.4 and Quant Center2.1 (3DHISTECH, Hungary).

### Statistical analysis

SPSS 23.0 software (IBM, America), and Graphpad Prim 7 (GarphPad Software, America) were used for statistical analysis. Student’s t-tests were used for comparing the differences between CDCA8, NF-YA, or other genes expression. Spearman correlations were applied to identify the relationship between CDCA8 with other potential interaction genes. Kaplan–Meier curves and cox regression models were used to determine the prognostic performance of patient survival. *p*-value < 0.05 was considered to be statistically significant.

## Results

### The potential critical hub genes in HCC progression by bioinformatics.

**Fig. 1:**
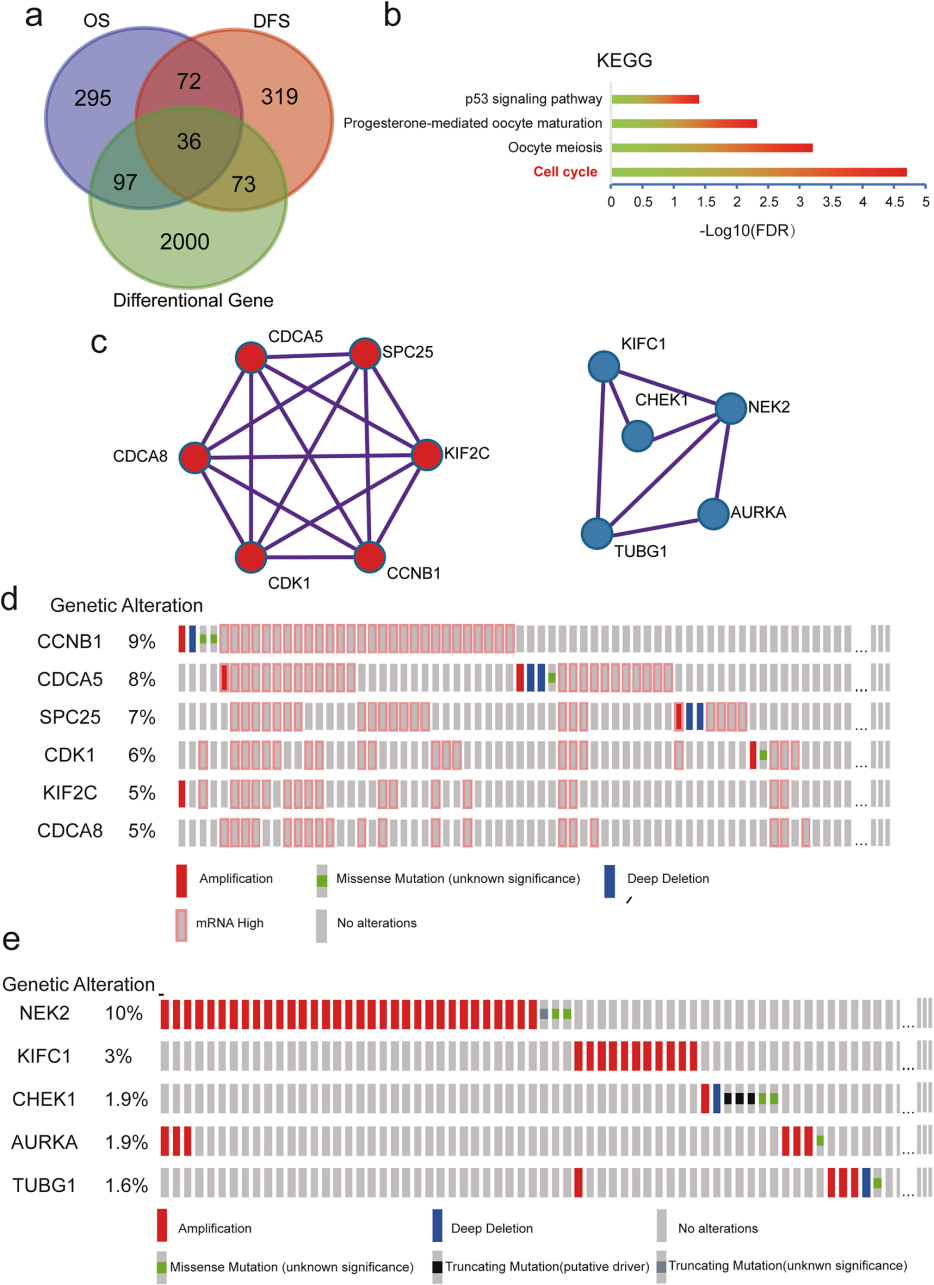
The identification of hub genes in the HCC by bioinformatic analysis. **a** The identification of 36 core genes were overlapped in the three groups. **b** KEGG enrichment analysis suggested that 36 core genes were primarily enriched in the cell cycle process. **c** The two hub genes were identified by MCODE analysis. **d** Hub gene I contained CCNB1, CDCA5, SPC25, CDK1, KIF2C, and CDCA8, with characterized by mRNA high **e** Hub gene II contained NEK2, KIFC1, AURKA, CHEK1, and TUBG1, with amplification features

To investigate novel oncogene drivers during HCC progression, we performed a systematic bioinformatics analysis using the GEPIA dataset. There were 2206 differentially expressed genes, 500 genes were related to overall survival, 500 genes were related to disease-free survival in the GEPIA dataset. As shown in Fig. 1a, a total of 36 genes overlapped in all three groups. Thus, we next applied Gene ontology (GO) and Kyoto Encyclopedia of Genes and Genomes (KEGG) analysis to explore the potential cellular mechanisms related to those 36 genes. GO analysis suggested that the 36 genes were significantly enriched for mitotic nuclear division, in chromosomal regions, and for histone kinase activity (Additional file 1: Fig. S1a–c). As shown in the Fig. 1b, KEGG analysis revealed that the cell cycle was the main pathway that the 36 genes involved. To narrow down candidate genes among the 36 genes, we used a MCODE analysis and found two core hub genes (Hub gene I and II) (Fig. 1c, Additional file 2: Fig. S2a, b). These two hub genes exhibited different characteristics (Fig. 1d, e). Hub gene I contained CCNB1, CDCA5, SPC25, CDK1, KIF2C, and CDCA8, was characterized by high level of mRNA, whereas hub gene II contained NEK2, KIFC1, AURKA, CHEK1, and TUBG1, had amplification features. After reviewing previous literature, we found that some genes, including CCNB1, CDCA5, SPC25, CDK1, KIF2C, have been reported to be involved in the progression of HCC [11, 20–23]. Although several previous papers have revealed the role of CDCA8 in HCC using bioinformatics analysis [24], experimental validation remains to be carried out, and the precise mechanism of CDCA8 in HCC development is still unknown. Thus, we chose to conduct an in-depth study of CDCA8’s mechanisms.

### Elevated CDCA8 levels are associated with a worse prognosis.

**Fig. 2:**
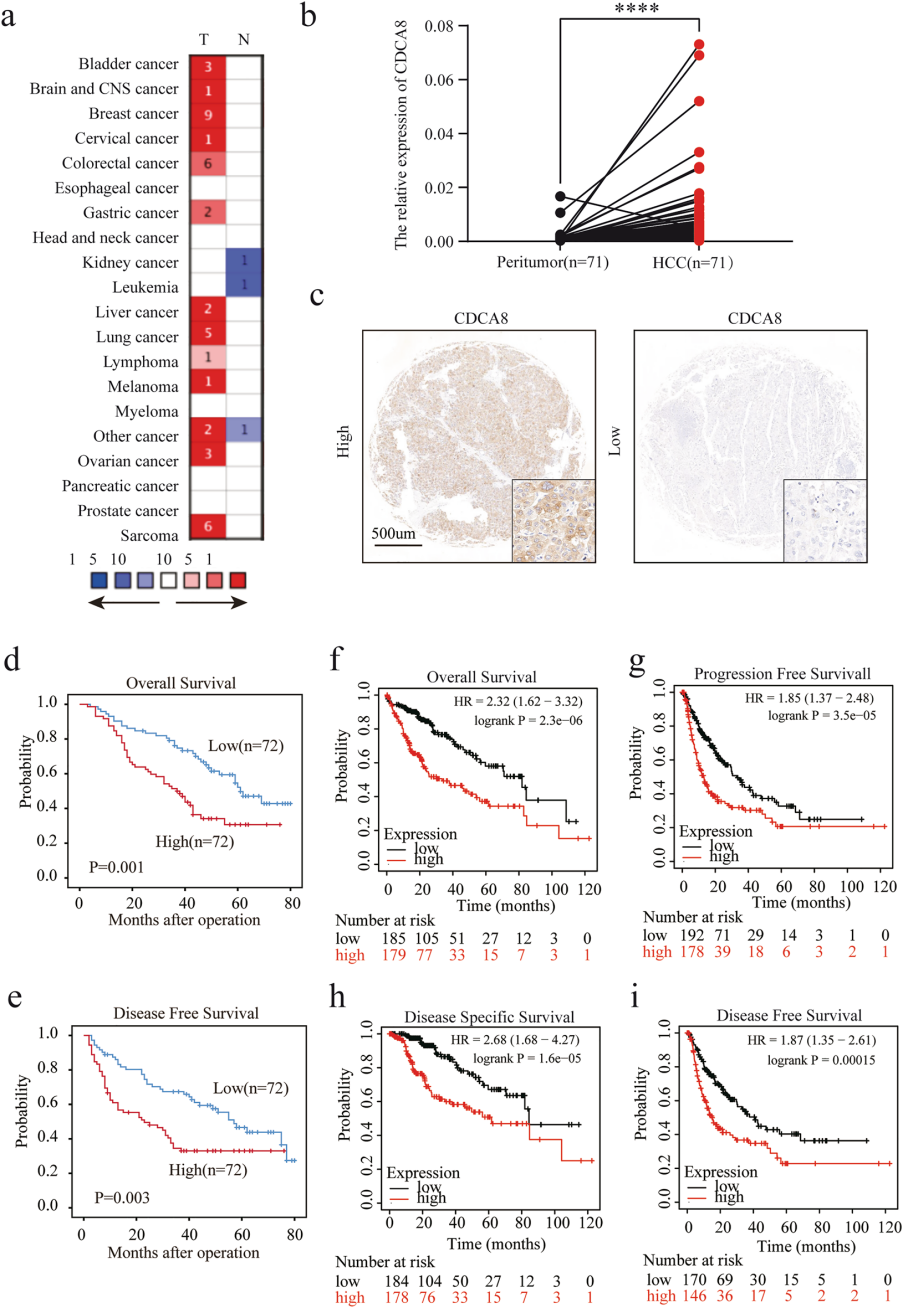
CDCA8 overexpression is associated with worse prognosis. **a** ONCOMINE datasets revealed that CDCA8 expression was higher in the tumor compared with normal tissues in multiple cancers. **b** The mRNA expression of CDCA8 in 71 pairs of HCC and peritumor tumors by qRT-PCR. **c** The CDCA8 level was detected by IHC in PKUSZ TMA including 144 HCC samples and typical pictures were presented. **d, e** Survival analysis of CDCA8 in 144 HCC samples indicted that the higher expression of CDCA8 was significantly related with **d** the overall survival and **e** disease-free survival. **f**–**i** K-M plotter (http://kmplot.com/analysis/) demonstrated that CDCA8 overexpression was remarkably associated with several different clinical outcomes. (****p < 0.0001)

To investigate the role of CDCA8 in HCC tissues, we examined CDCA8 mRNA level, which were up-regulated in multiple cancers, including liver cancer, bladder cancer, colorectal cancer, and gastric cancer in the ONCOMINE dataset (Fig. 2a). Subsequently, CDCA8 expression was evaluated using qRT-PCR in our HCC cohorts. CDCA8 levels were elevated in cancer tissues compared to adjacent normal tissues (Fig. 2b, *p *< 0.0001, n = 71). We also performed immunohistochemical analysis on PKUSZ TMA. 144 cases were stratified into two subgroups based on median CDCA8 expression (High, n = 72; Low, n = 72) (Fig. 2c). A chi-square analysis was used to compare the CDCA8 levels and clinicopathological features. The results showed that CDCA8 levels were significantly positively associated with AFP levels (*p* = 0.003), tumor number (*p* = 0.007), and vascular invasion (*p* = 0.045) (Table 1). Kaplan–Meier survival curves indicated that the patients with higher CDCA8 levels had remarkably shorter overall survival (OS) and disease-free survival (DFS) rates (Fig. 2d, e). Furthermore, we found that high CDCA8 levels predicted shorter OS, shorter progression-free survival, shorter disease-specific survival, and worse DFS in public datasets (Fig. 2f–i). Finally, multivariate cox analysis showed that CDCA8 was an independent prognostic marker for both OS and DFS (Table 2). Taken together, the above results showed that the expression of CDCA8 is increased in HCC tissues, and acts as an oncogene, leading to worse survival outcomes.

**Table 1:** Correlation between CDCA8 expression and clinical characteristics in HCC (n = 144)

Clinical characteristics	CDCA8 expression		p value
	Low (N = 72)	High (N = 72)	
Age, year (≤ 50 vs. > 50)			
≤ 50	31	37	0.317
> 50	41	35	
Sex (Female vs. Male)			
Female	10	9	0.806
Male	62	63	
HBsAg (Negative vs. Positive)			
Negative	10	11	0.813
Positive	62	61	
AFP, ng/mL (≤ 13.4 vs. > 13.4)			
≤ 13.4	35	18	**0.003**
> 13.4	37	54	
Liver cirrhosis (Negative vs. Positive)			
Negative	11	11	1.000
Positive	61	61	
Tumor size, cm (≤ 5 vs. > 5)			
≤ 5 cm	40	36	0.504
> 5 cm	32	36	
Tumor number (Single vs. Multiple)			
Single	61	47	**0.007**
Multiple	11	25	
Vascular invasion (Negative vs. Positive)			
Negative	45	33	**0.045**
Positive	27	39	
Tumor encapsulation (Complete vs. None)			
Complete	51	47	0.475
None	21	25	
Tumor differentiation (I + II vs. III + IV)			
I + II	48	44	0.488
III + IV	24	28	
TNM stage (I vs. II + III)			
I–II	43	18	0.813
III	29	54	

Bold values indicate *p* < 0.05

**Table 2:** Univariate and multivariate analyses of prognostic factors with OS and DFS in HCC (n = 144)

Variable	OS		DFS	
	HR (95%CI)	p	HR (95%CI)	p
Univariate analysis				
Age, year (≤ 50 vs. > 50)	0.735 (0.472–1.145)	0.174	0.771 (0.502–1.185)	0.236
Sex (Female vs. Male)	1.431(0.688–2.974)	0.338	1.368(0.684–2.736)	0.376
HBsAg (Negative vs. Positive)	1.299 (0.649–2.602)	0.460	1.090 (0.577–2.058)	0.791
AFP, ng/mL (≤ 13.4 vs. > 13.4)	1.556 (0.971–2.495)	0.066	1.645 (1.038–2.608)	**0.034**
Liver cirrhosis (No vs. Yes)	1.651 (0.824–3.310)	0.157	1.563 (0.806–3.031)	0.186
Tumor size, cm (≤ 5 vs. > 5)	1.566 (1.005–2.441)	**0.047**	2.094 (1.355–3.235)	**0.001**
Tumor number (Single vs. Multiple)	1.938 (1.209–3.106)	**0.006**	2.012 (1.263–3.207)	**0.003**
Vascular invasion (Negative vs. Positive)	2.147 (1.370–3.364)	**0.001**	1.908 (1.236–2.945)	**0.004**
Tumor encapsulation (Complete vs. None)	1.785 (1.132–2.815)	**0.013**	1.817 (1.172–2.819)	**0.008**
Tumor differentiation (I + II vs. III + IV)	1.757 (1.124–2.748)	**0.013**	1.786 (1.155–2.761)	**0.009**
TNM stage (I vs. II + III)	4.082 (2.420–6.886)	**0.000**	3.214 (1.982–5.212)	**0.000**
CDCA8 (Low vs. High)	2.104 (1.334–3.32)	**0.001**	1.927 (1.241–2.991)	**0.003**
Multivariate analysis				
AFP, ng/mL (≤ 13.4 vs. > 13.4)	NA	NA	1.230 (0.754–2.007)	0.407
Tumor size, cm (≤ 5 vs. > 5)	1.506 (0.941–2.41)	0.088	2.192 (1.382–3.476)	**0.001**
Tumor number (Single vs. Multiple)	1.649 (1.011–2.691)	**0.045**	1.663 (1.01–2.738)	**0.046**
Vascular invasion (Negative vs. Positive)	1.675 (1.017–2.756)	**0.043**	1.383 (0.853–2.241)	0.189
Tumor encapsulation (Complete vs. None)	1.300 (0.782–2.161)	0.311	1.266 (0.773–2.073)	0.348
Tumor differentiation (I + II vs. III + IV)	1.755 (1.115–2.762)	**0.015**	1.797 (1.152–2.802)	**0.010**
CDCA8 (Low vs. High)	1.903 (1.174–3.083)	**0.009**	1.778 (1.113–2.839)	**0.016**

Bold values indicate *p* < 0.05

### CDCA8 promotes HCC growth and colony formation in vitro

**Fig. 3:**
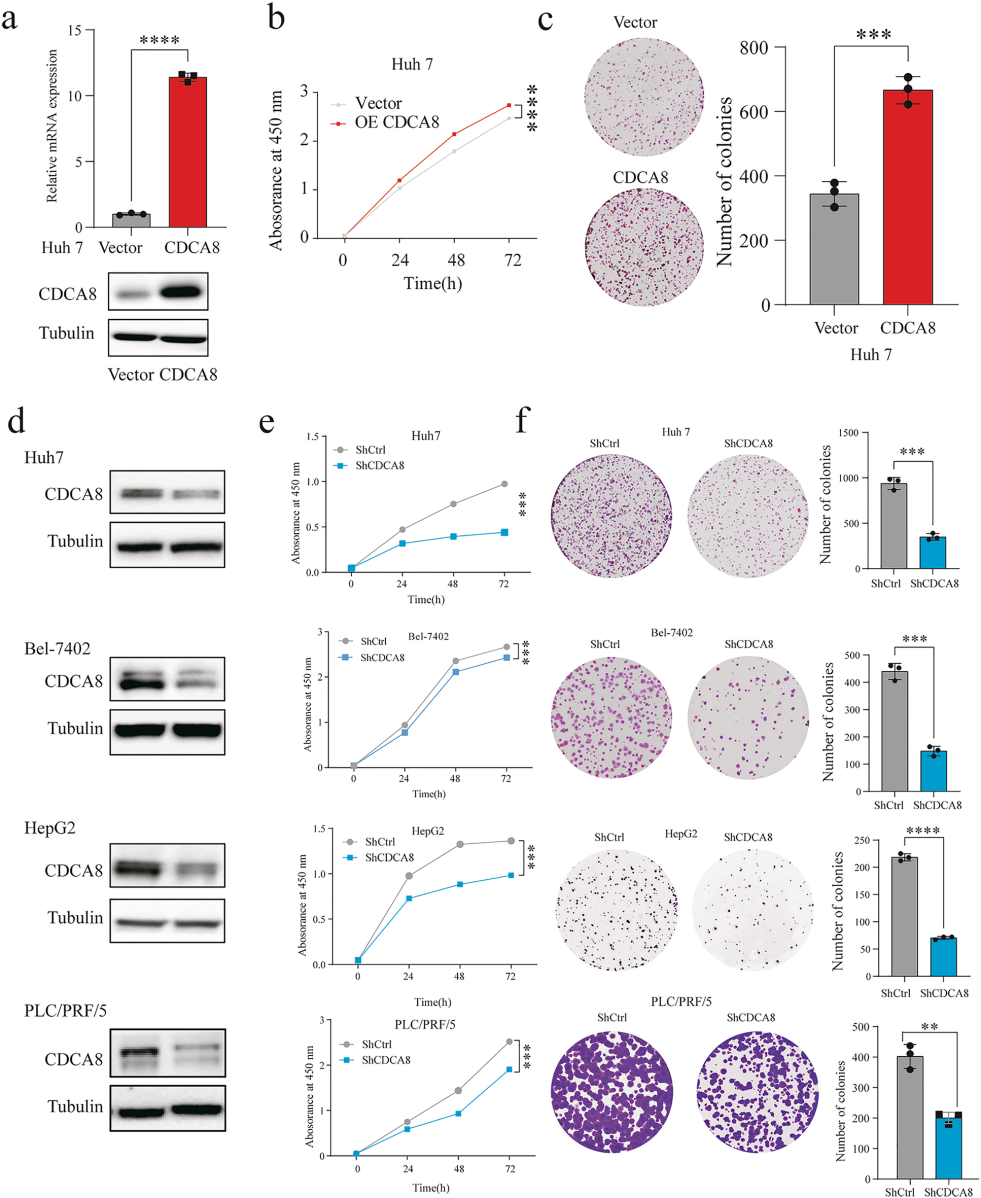
CDCA8 promotes HCC cell proliferation in vitro. **a** The efficiency of transfection in Huh7 cell line were confirmed by qRT-PCR and Western blot. **b**, **c** CDCA8 overexpression in Huh7 cells promotes cell proliferation by **b** CCK-8 and **c** colony formation assays. **d** Inhibition expression of CDCA8 in Huh7, Bel-7402, HepG2, and PLC/PRF/5 was confirmed by Western blot. **e, f** Inhibition expression of CDCA8 in Huh7, Bel-7402, HepG2, and PLC/PRF/5 cells delayed cell proliferation by **e** CCK-8 and** f** colony formation assays. (**p < 0.01; ***p < 0.001; ****p < 0.0001)

As the correlation between CDCA8 and patient survival had been validated, we then explored CDCA8’s role in cell proliferation. The level of CDCA8 was overexpressed in Huh7 cell lines by lentivirals (Fig. 3a). We observed that CDCA8 overexpression enhanced proliferation capacity in Huh7 using CCK-8 and colony formation assays (Fig. 3b, c). Western blot analysis showed that shCDCA8 caused protein loss (Fig. 3d). As expected, inhibition of CDCA8 remarkably decreased the proliferative abilities and the number of colonies in shCDCA8 cells (Fig. 3e, f). Moreover, we performed the EdU assays and observed that the ratios of EdU-positive cells in the CDCA8-shRNA cells were lower than those of control cells (Additional file 3: Fig. S3). The results demonstrated that CDCA8 overexpression promoted cell viability, colony formation capacity, and DNA synthesis.

### CDCA8 enhances the invasiveness of HCC cells through the MEK/ERK signaling

**Fig. 4:**
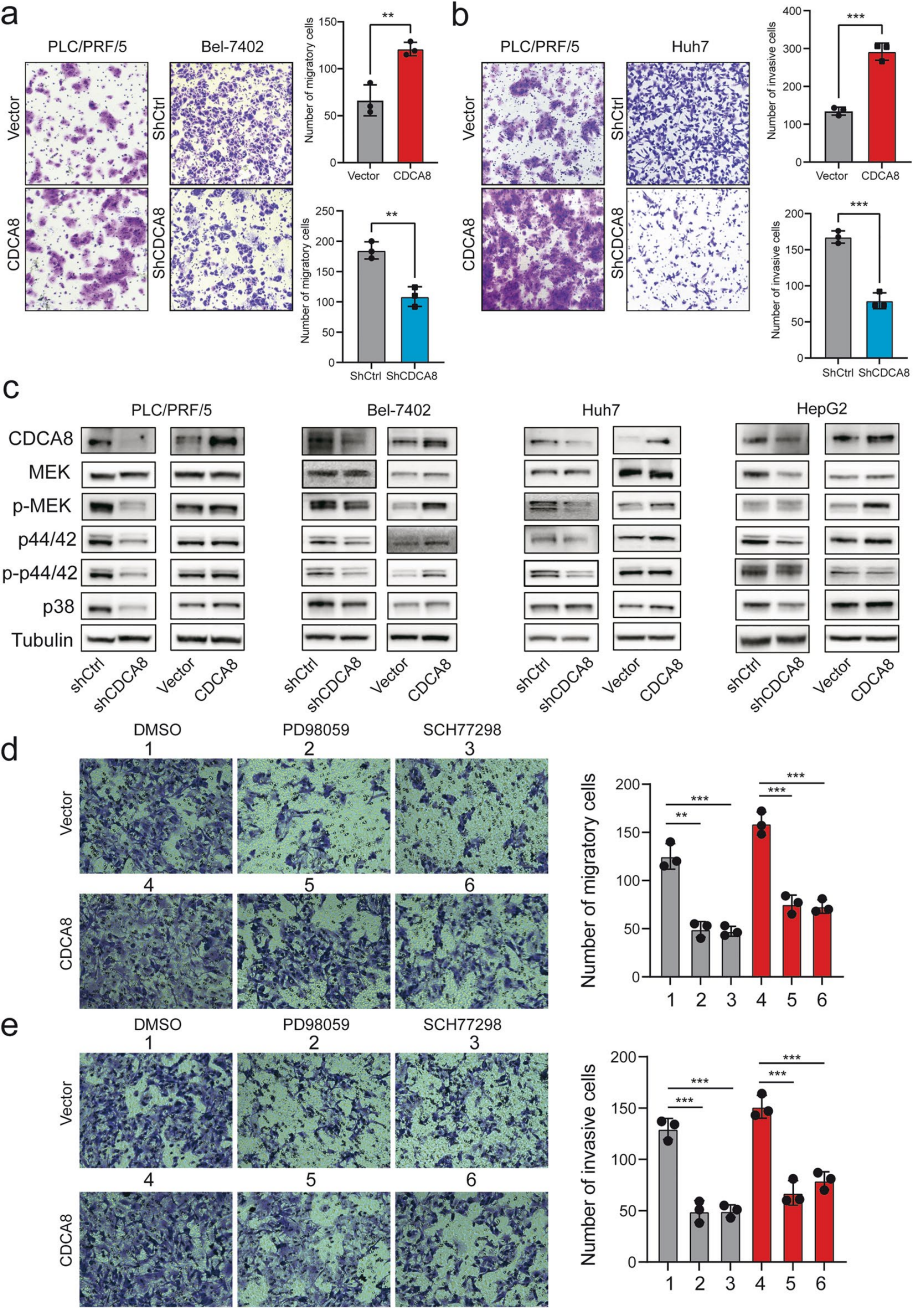
CDCA8 promotes cell migration and invasion of HCC cells in vitro. **a** Transwell migration assay revealed that CDCA8 overexpression promote the migration capability of PLC/PRF5 cells while CDCA8 inhibition in Bel-7402 cells showed the opposite effect. **b** Transwell invasion assay revealed that CDCA8 overexpression promoted the invasion capability of PLC/PRF5 cells while CDCA8 knockdown in Huh7 cells exhibited the opposite result. **c** The expression levels of MAPK pathway related-proteins were assessed after CDCA8 increased or decreased in four HCC cell lines. **d, e** Representative pictures of (**d**) migration and (**e**) invasion assays with or without PD98059 or SCH77298 in HepG2 cells. (**p < 0.01; ***p < 0.001)

We carried out Transwell without Matrigel experiments to further assess CDCA8-mediated migratory patterns. These studies revealed that the number of migrating cells was remarkably increased in PLC/PRF/5 CDCA8-overexpressing cells, whereas the number of migrating cells was decreased in Bel-7402 shCDCA8 cells (Fig. 4a). Similarly, Transwell with Matrigel assays showed that the number of invading cells in PLC/PRF/5-CDCA8 cells was remarkably higher compared to control cells, while the number of invading cells in Huh7-shCDCA8 cells was lower compared with control cells (Fig. 4b).

To further evaluate the molecular mechanism underlying the CDCA8-mediated HCC phenotype, we investigated mitogen-activated protein kinase (MAPK) pathway markers in CDCA8-knockdown and CDCA8 overexpression cells. Immuno-blots suggested that CDCA8 deficiencies suppressed the phosphorylation of MEK and ERK significantly in four HCC CDCA8-knockdown cells (Fig. 4c). Similarly, CDCA8 overexpression induced MEK and ERK phosphorylation in four HCC CDCA8-overexpressing cells (Fig. 4c). We also assessed p38 (a key factor in the MAPK pathway) level, after CDCA8 up- and down-regulation. Western blot results showed that p38 expression was inconsistent across four cell lines (Fig. 4c). Furthermore, we used PD98059 and SCH772984 to inhibit of the MEK/ERK signaling pathway. We also evaluated the migration and invasion assays in the CDCA8 overexpression and control cells treated with or without PD98059 and SCH77298. As expected, repression of activated MEK1/2 and ERK1/2 successfully reversed the malignant behaviors of HepG2 cells caused by overexpression of CDCA8 (Fig. 4d, e). Taken together, these results suggested that CDCA8 enhances the tumor growth and invasiveness of HCC cells via the MEK/ERK pathway.

### CDCA8 promotes HCC proliferation and pulmonary metastasis in vivo

**Fig. 5:**
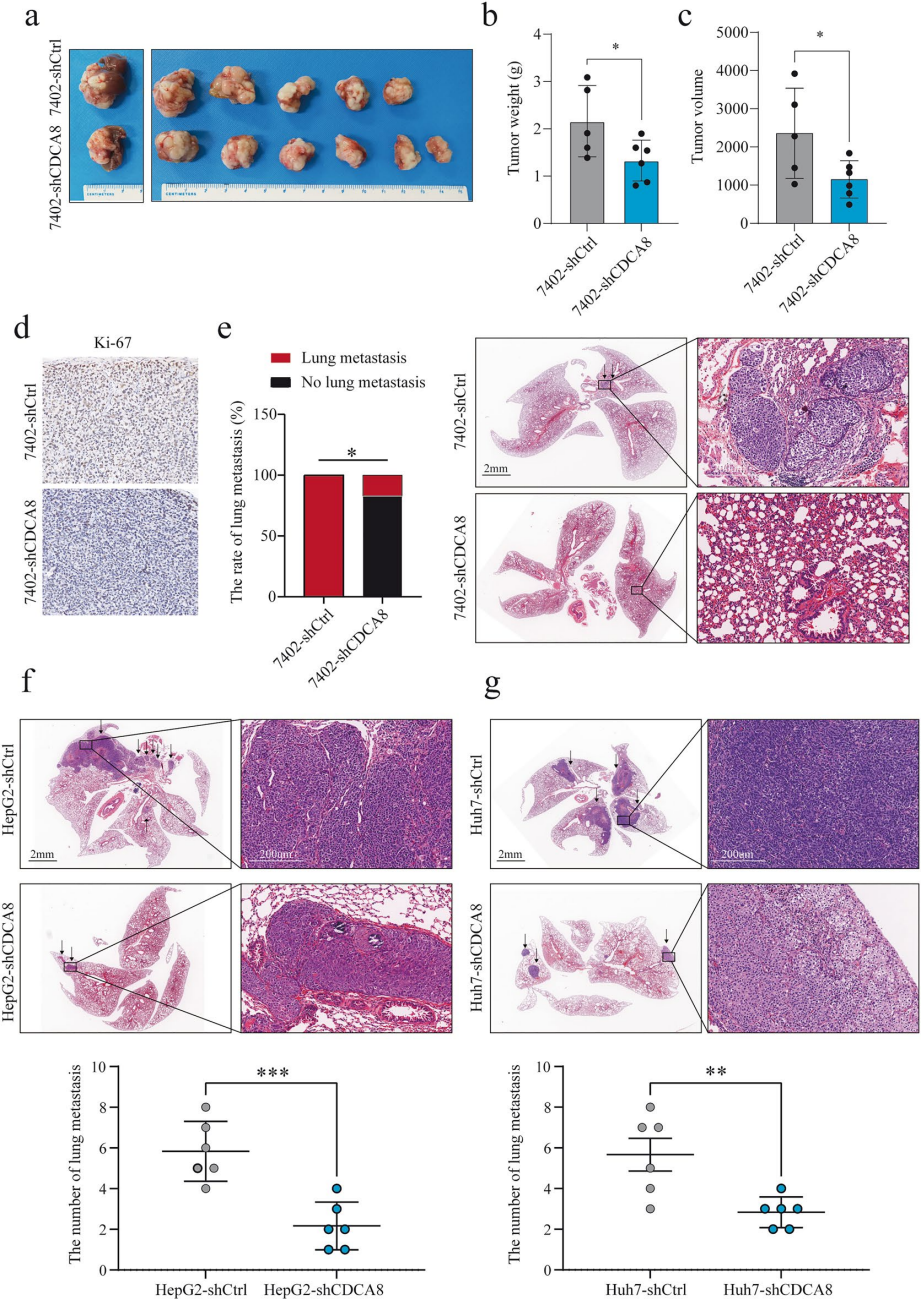
Knockdown of CDCA8 inhibited tumor growth and pulmonary metastasis in vivo. **a** Bel-7402 cell with different CDCA8 expression were subcutaneously transplanted the xenograft into BALB/c nude mice (n = 6 for shCDCA8; n = 5 for shCtrl) **b** Tumor weights and **c** tumor sizes were measured. **d** Tumors were stained by Ki-67. **e** The number of lung metastasis in different groups were measured. **f** HepG2 and **g** Huh7 cells with different CDCA8 expression were injected into BALB/c nude mice (n = 6 for each group) through the tail vein to establish lung metastasis models. (*p < 0.05; **p < 0.01; ***p < 0.001)

To investigate the biological function of CDCA8 in HCC mouse models, Bel-7402 cells with CDCA8 knock down (shCDCA8) or shCDCA8 control (shCtrl) cells were injected into immunodeficient nude mice to form the subcutaneous tumors. Then, small pieces of subcutaneous tumors were transplanted into the livers of new immunodeficient nude mice. The data suggested that the tumor weights and sizes in the Bel-7402-shCDCA8 cells were significantly lower than in the shCtrl group (Fig. 5a–c). Further, immunohistochemical staining for Ki-67 showed that CDCA8 promoted tumor growth in vivo, corroborating the proliferative effects of CDCA8 in HCC (Fig. 5d). Of note, pulmonary metastases were observed in 100% (5/5) of shCtrl group cells, whereas lung metastasis was found in only one (1/6, 16.7%) shCDCA8 group mouse (Fig. 5e).

We also constructed tail vein models in HepG2-shCDCA8 and Huh7-shCDCA8 and negative controls to evaluate the effects of CDCA8 on pulmonary metastasis. The results of HE examinations of lung tissues revealed that sections from the HepG2-shCDCA8 and Huh7-shCDCA8 exhibited lower lung metastatic nodules compared with the control group (Fig. 5f, g). Overall, our results demonstrated that CDCA8 overexpression promoted pulmonary metastasis in vivo.

### RNA-sequencing reveals novel downstream targets and pathways related to CDCA8

**Fig. 6:**
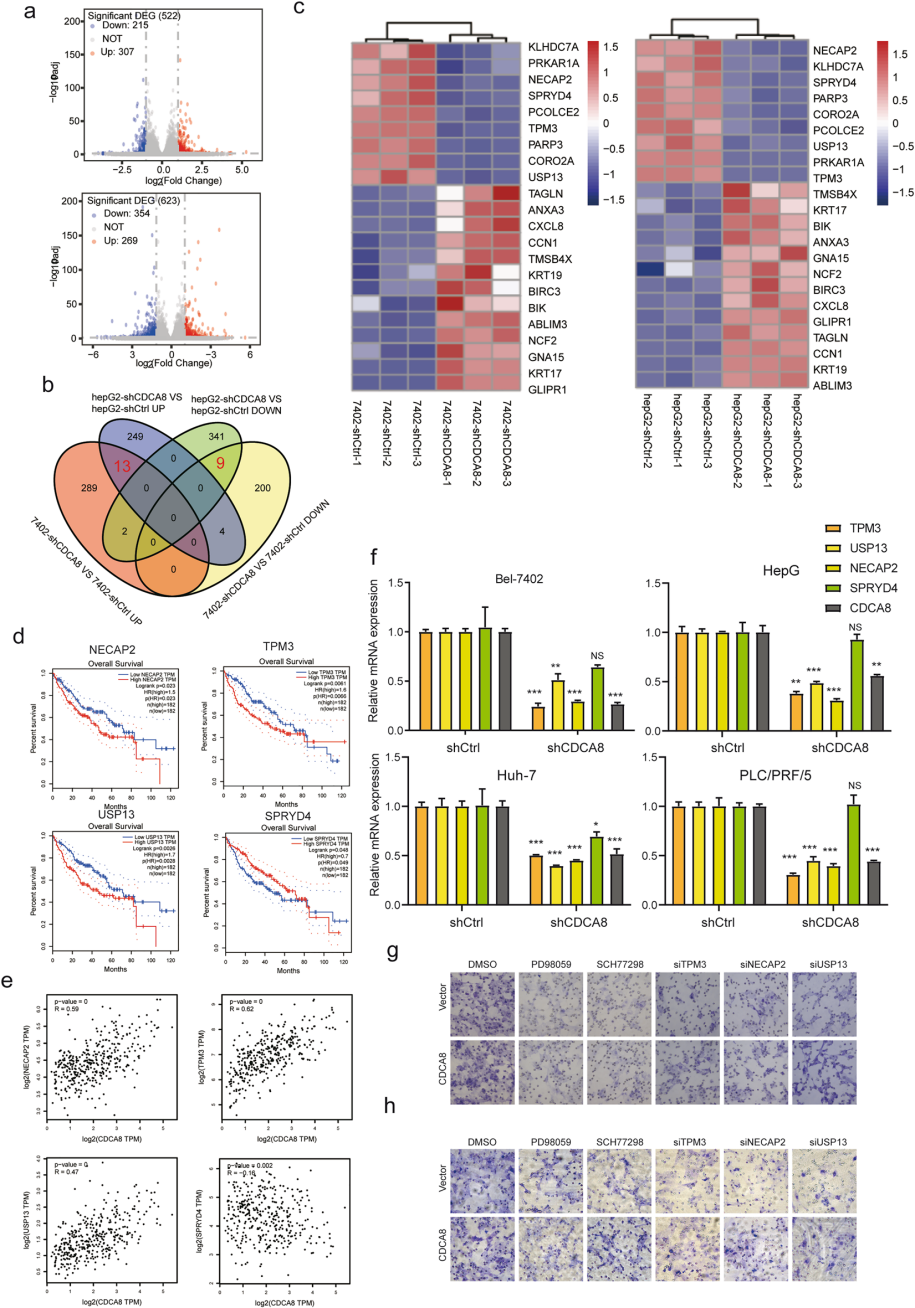
CDCA8 mediates the expression of TPM3, NECAP2, and USP13 in HCC cells. **a** The volcano map. **b** Veen diagram **c** The heatmap **d** Kaplan–Meier analysis was used to assess the effect of TPM3, NECAP2, USP13, and SPRYD4 on HCC patient survival. **e** The scatter plot were used to describe the relationship between CDCA8 and TPM3, NECAP2, USP13, SPRYD4 in HCC samples. **f** qRT-PCR were performed in four cell lines with CDCA8 inhibition. **g, h** Representative pictures of (**g**) migration and (**h**) invasion assays with or without PD98059 or SCH77298 or siRNA targeting TPM3, NECAP2, and USP13 in PLC/PRF/5 cells. (*p < 0.05; **p < 0.01; ***p < 0.001; ****p < 0.0001)

To investigate potential novel downstream targets of CDCA8 in HCC cells, we used the Next- Generation sequencing to complete the downstream gene profiling in Bel-7402-shCDCA8 and HepG2-shCDCA8 cell lines compared to negative controls. Compared to the control group, a total of 215 genes showed decreases and 307 genes showed increases in the Bel-7402-shCDCA8 cells, while 354 genes showed decreases and 269 genes had increased in HepG2-shCDCA8 cells (Fig. 6a). Veen diagrams showed that 13 genes were co-elevated and 9 genes co-decreased were in the different cell lines (Fig. 6b). As shown in Fig. 6c, a heatmap revealed that USP13, CORO2A, PRKAR1A, TPM3, NECAP2, PCOLCE2, SPRYD4, PARP3, and KLHDC7A were down-regulated in the Bel-7402-shCDCA8 and HepG2-shCDCA8 lines, while BIRC3, GNA15, BIK, NCF2, KRT17, ANXA3, GLIPR1, CCN1, TAGLN, CXCL8, KRT19, ABLIM3, and TMSB4X were up-regulated in the Bel-7402-shCDCA8 and HepG2-shCDCA8 cell lines (Fig. 6c). Among these down-regulated candidate genes, four genes (TPM3, NECAP2, USP13, SPRYD4) could predict the OS of HCC patients (Fig. 6d). We also examined the relationship between CDCA8 and the four genes were investigated in the TCGA data. CDCA8 mRNA levels were positively associated with TPM3 (R = 0.62, *p* = 0), NECAP2 (R = 0.59, *p* = 0), and USP13 (R = 0.47, *p* = 0), but negatively associated with SPRYD4 (R = − 0.16, *p* = 0.002) (Fig. 6e). qRT-PCR was further performed to validate these findings of the four targets. TPM3, NECAP2, and USP13 were significantly down-regulated in the Bel-7402-shCDCA8, HepG2-shCDCA8, Huh7-shCDCA8, and PLC/PRF/5-shCDCA8 cell lines, whereas SPRYD4 expression was not found to be significant inhibited in the four cell lines (Fig. 6f). Furthermore, we used PD98059 and SCH772984 to inhibit of the MEK/ERK signaling pathway and siRNA targeting TPM3, NECAP2, and USP13 to perform the rescue experiments. We performed the migration and invasion assays in the CDCA8 overexpression and control cells treated with or without TPM3, NECAP2, and USP13. As expected, inhibition of MEK/ERK pathway or repression of TPM3, NECAP2, and USP13 successfully reversed the malignant behaviors of PLC/PRF/5 cells caused by overexpression of CDCA8 (Fig. 6g, h). Thus, CDCA8 promoted HCC proliferation and invasion by up-regulating TPM3, NECAP2, and USP13 via MEK/ERK pathway.

### High NF-YA as well as NF-YA levels combined with CDCA8 predict poor outcome

**Fig. 7:**
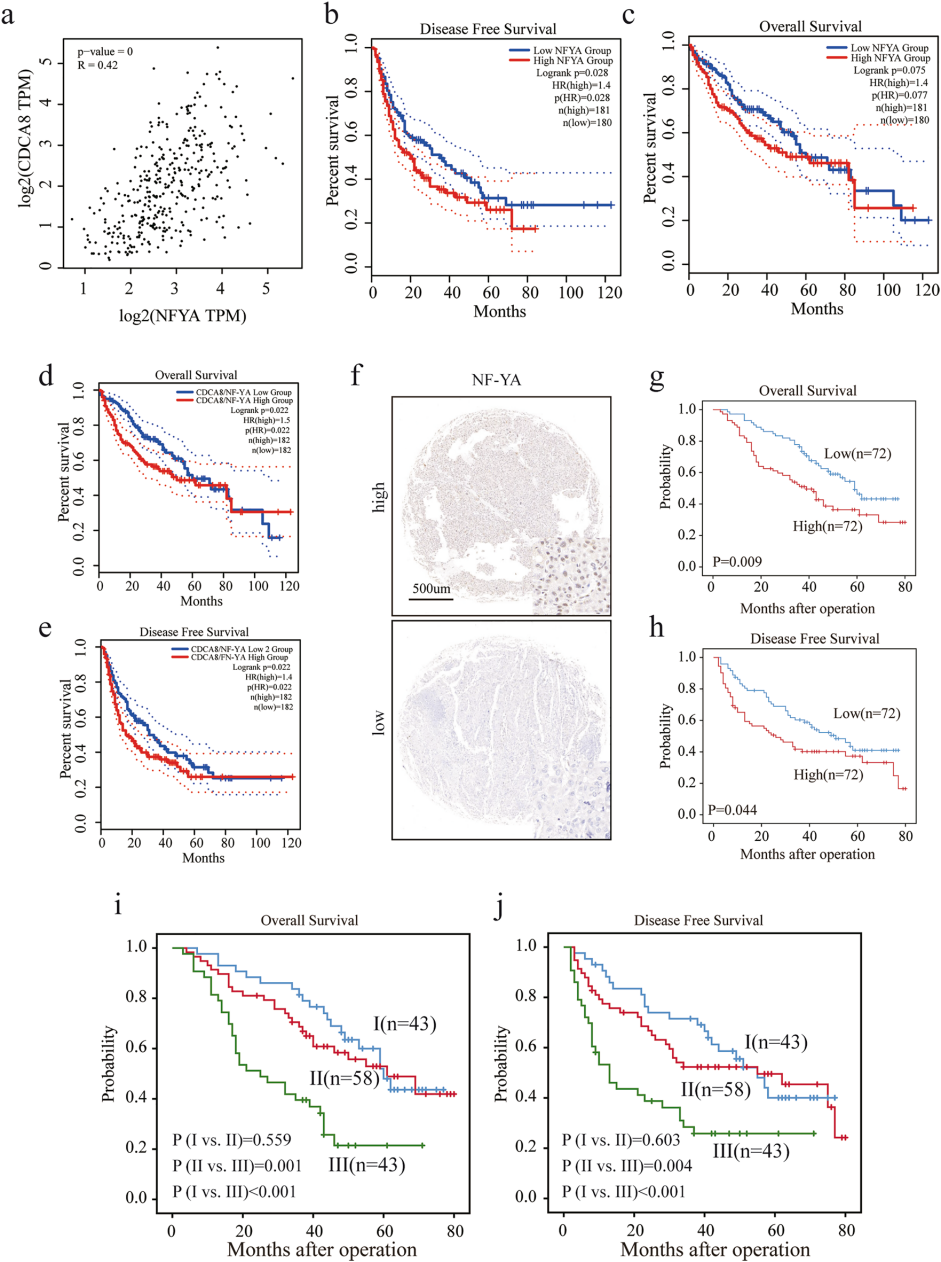
High NF-YA alone or in combined with CDCA8 predict poor outcome. **a** The scatter blot showing the relationship between CDCA8 and NF-YA expressions using data from TCGA, with Spearman correlation coefficient. **b, c** The expression of NY-YA was associated with **b** DFS and **c** OS. **d, e** High expression of NF-YA combined with CDCA8 predicted poor **d** OS and **e** DFS. **f** Immunohistochemical analysis of NF-YA in TMA including 144 clinical samples. **g, h** The level of NY-YA was significantly with poor **g** OS and **h** DFS. **i, j** Prognostic values of CDCA8 combined with NF-YA expression. I, CDCA8^Low^/NF-YA^Low^; II, CDCA8^Low^/NF-YA^High^ and CDCA8^High^/NF-YA^Low^; III, CDCA8^High^/NF-YA^High^

To further investigate the mechanism responsible for CDCA8 overexpression in HCC, we found that there was a significantly correlation between NF-YA levels and the CDCA8 levels in the TCGA dataset (Fig. 7a). In addition, Kaplan Meier results based on TCGA suggested that NA-YA level were remarkably positively correlated with DFS in HCC patients (Fig. 7b, p = 0.028), whereas high NF-YA expression was associated with shorter OS (although only at trend level; Fig. 7c, p = 0.075). We also observed that CDCA8 combined with NF-YA levels were strong predictors for patients’ prognosis (Fig. 7d, e). We next performed an immunohistochemical staining analysis of TMA (Fig. 7f) and observed that NF-YA levels were significantly associated with shorter OS and DFS (Fig. 7g, h). The median OS and DFS were 48.5 and 44 months for cases in NF-YA^Low^ subgroup as compared with 36 months and 21 months for cases in NF-YA^High^ group. To explore the combined effects of NF-YA and CDCA8 on the HCC survival, we stratified 144 cases into three subgroups according to the density expression of NF-YA and CDCA8: I, CDCA8^Low^/NF-YA^Low^; II, CDCA8^Low^/NF-YA^High^ and CDCA8^High^/NF-YA^Low^; III, CDCA8^High^/NF-YA^High^. Notably, patients with in subgroup III exhibited the worse OS and DFS (Fig. 7i, j).

### NF-YA/CDCA8 axis promotes HCC growth and metastasis.

**Fig. 8:**
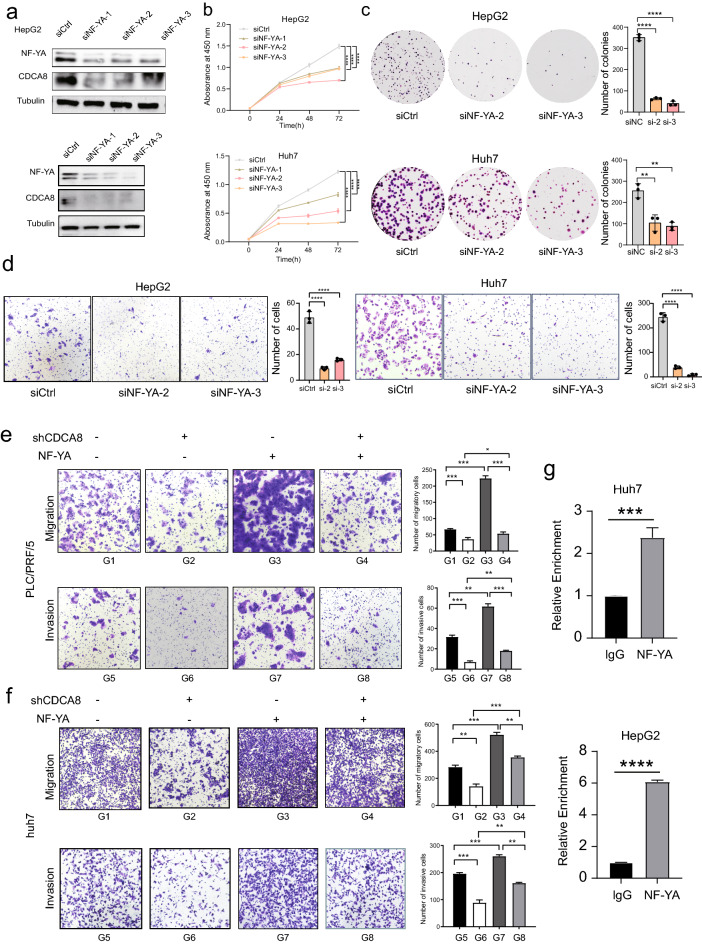
NF-YA/CDCA8 axis promotes HCC growth and metastasis. **a** CDCA8 and NF-YA levels were evaluated in HCC cell lines transfected with the three siRNA for NF-YA. **b** CCK-8 and **c** The colony survival assay were performed. **d** Measurement of cell migration by transwell assay using cell describe in **a**. **e**, **f** Measurement of cell migration by transwell assays using HepG2 and Huh7 cells with NF-YA or vector, and inhibition of CDCA8 in NF-YA overexpressing cells. **g** The relative enrichment of NF-YA and lgG in Huh7 and HepG2 cells. (*p < 0.05; **p < 0.01; ***p < 0.001; ****p < 0.0001)

As shown in Fig. 8a, we observed that CDCA8 expression was suppressed after three siRNA for NF-YA in HepG2 and Huh7 cell lines. In vitro experiments suggested that the loss of NY-FA significantly suppressed HepG2 and Huh7 cells’ proliferation capacities (Fig. 8b, c). Furthermore, HepG2 and Huh7 cells’s migratory abilities were inhibited by NF-YA knockdowns (Fig. 8d). To investigate the role of NF-YA/CDCA8 axis in the progression of HCC, we performed rescue experiments in HCC cell lines. CDCA8 loss could attenuate cell migration and invasiveness that was enhanced by the forced expression of NF-YA in PLC/PRF/5 cells (Fig. 8e). Similar results were observed in Huh7 cell lines (Fig. 8f). Lastly, we performed the CHIP-PCR experiments and found that NF-YA bind to CDCA8 promoter, indicating that NF-YA directly regulate CDCA8 expression (Fig. 8g). The above results suggested that CDCA8 is a direct downstream gene of NF-YA. Therefore, this regulatory relationship of NF-YA and CDCA8 is direct.

## Discussion

Several recent reports have suggested that CDCA family members are involved in tumor proliferation and development. A comprehensive bioinformatic analysis of gene expression in the TCGA dataset showed that overexpression of CDCA8 is an independent risk factor for patient prognosis. These findings were also validated in several GEO datasets and our HCC cohort. Previous studies [25, 26] have found that CDCA8 is closely related to OS and DFS of HCC patients, whereas we observed that CDCA8 expression is not only associated with poorer OS and DFS, but also poorer progression free survival and disease specific survival. On the other hand, previous literatures [25, 26] revealed that CDCA8 affects liver cancer progression through E2F1 and ATF3. In our study, TPM3, NECAP2, and USP13 were validated as novel downstream targets of CDCA8 by RNA-sequencing and in vitro experiments. Moreover, the rescue experiments demonstrated that TPM3, NECAP2, and USP13 knockdown attenuate CDCA8-mediated cell migration and invasion in vitro. Lastly, we reported that NF-YA is an upstream regulator of CDCA8. Summarily, on the basis of previous study, we expanded the understanding of the role of CDCA8 in HCC development.

Previous studies have showed that CDCA8 overexpression drives malignant tumor behavior, growth, invasion, and metastasis in many types of cancers. CDCA8 loss has been shown to decrease the malignant behavior of melanomas through the ROCK signaling pathway [27]. CDCA8 silencing also suppressed cell growth, migration, and invasiveness in gliomas [28]. CDCA8 expression has also been shown to be involved in several signatures, such as hypoxia-related signature [29], metastasis-related mRNAs [12], and glycolysis-related genes [30], which could also predict HCC diagnosis and prognosis, and may reflect different tumor immune or metabolic microenvironments. CDCA8 was reported to be an independent prognostic factor for liver cancer [31]. Similar to previous studies, we found that the CDCA8 levels was highly increased in the multiple cancer types, including HCC. Additionally, CDCA8 overexpression was positively correlated with AFP levels, increased tumor numbers, vascular invasion, and shorter survival, indicating that CDCA8 is involved in the malignant behavior of HCC and is correlated with worse outcomes for HCC patients. Multivariate Cox model analysis showed that high CDCA8 levels were independent risk predictors in HCC. We found that CDCA8 expression was up-regulated in multiple cancers. CDCA8 strongly promoted cellular proliferation and metastasis in vitro and in vivo. Furthermore, CDCA8 knockdown significantly suppressed p-MEK and p-ERK in the MAPK pathway in HCC cells. The MAPK pathway is one of the well-known signaling cascades in cancer research. A number of cancer phenotypes, including cellular proliferation, invasion, survival, and apoptosis were mediated by MAPK pathway [32]. p38, JNK and the ERK are the main players in the MAPK pathway that regulate the growth, and survival of various tumors [33]. In addition to MEK/ERK molecular factors, we also assessed the expression of p38 after overexpressing/inhibiting CDCA8 expression, and found inconsistent p38 protein levels across four cell lines. Thus, we concluded that CDCA8 enhanced the growth and invasiveness of HCC cells via the MEK/ERK pathway.

We also performed RNA-sequencing and found many potential downstream targets of CDCA8. TPM3, NECAP2, and USP13 expression were inhibited by CDCA8 knockdown in four HCC cell lines, suggesting that they were likely downstream molecules. Previous literature had reported that TPMs have critical functions during tumor progression. TPM3 fused with ALK to promote hematopoietic tumorigenesis [34]. Similarly, TPM3 accelerated leukemia via fusion with PDGFRB. TPM3 also leaded to papillary thyroid carcinoma via rearrangement with NTRK1 [35]. TPM3 overexpression induced epithelial-mesenchymal transition in HCC [36]. USP13 was involved in the occurrence and progression of cancer. USP13 promoted HCC cell growth and metastasis by regulating the TLR4/MyD88/NF-κB pathway [37]. Additionally, Yue Wu et al. showed that USP13 expression was up-regulated in non-small cell lung cancer, and drove cancer development via the AKT/MAPK pathway [38]. The above results that CDCA8 may activate MAPK signaling through mediating USP13 levels. Previous studies revealed that NECAP2 regulated endocytic recycling of EGFR [39]. EGFR activation was closely correlated with tumor proliferation, invasion, metastasis and chemoresistance [40]. However, the relationship between NECAP2 and CDCA8 remains unknown. Further studies are needed to explore the mechanism underlying the relationship between TPM3, NECAP2, USP13, and CDCA8.

As for potential upstream targets of CDCA8, previous literature had suggested that the CDCA8 was transcriptional promoted by NF-Y in cancer cells [14]. NF-Y is a ubiquitous heterotrimeric TF [41] with three isoforms: NF-YA, NF-YB, and NF-YC [15]. NF‐YA functions as an oncogene or suppressor depending on the specific tumor types [42]. NF-YA levels were remarkably correlated with CDCA8 levels. We observed that NF-YA could suppress the production of CDCA8, and that NF-YA knockdown suppressed cell proliferative and migratory abilities. Previous studies showed that NF-YA overexpression was correlated with the stem cell markers KLF4, and SALL4, as well as cell-cycle genes [43]. We hypothesized that NF-YA could regulate cell-cycle genes through the mediation of CDCA8 expression. Additionally, the results of clinical samples from our center showed that NF-YA expression is an independent and reliable risk factor for patients with HCC. Strikingly, patients with the NF-YA^high^/CDCA8^high^ features were more likely to suffer tumor recurrence and have had outcomes after tumor resection surgery.

## Conclusions

The high death rate amongst HCC patients is a major concern, and the underlying molecular mechanisms are incompletely understood. The present work demonstrates that the NF-YA/CDCA8 axis promote HCC proliferation and invasion via the MEK/ERK pathway. Overexpression of CDCA8 is a reliable predictor of tumor malignancy and a worse clinical prognosis. In summary, CDCA8 combined with NF-YA could serve as strong therapeutic targets for HCC patients.

## Supplementary Information

**Additional file 1: Figure S1.** Gene ontology enrichment analysis of 36 genes. a Mitotic nuclear division was main annotation in the biological process category. b Chromosomal region was main annotation in the cellular component category. **c** Histone kinase activity was main annotation in the molecular function category.

**Additional file 2: Figure S2.** The network of 26 genes. a The pathway underlying 36 genes. b MCODE analysis revealed that two core hub genes among 36 genes.

**Additional file 3: Figure S3.** HepG2, PLC/PRF/5 and Huh7 cells were seeded onto coverslips and DNA synthesis was assessed via EdU immunofluorescence staining. (*, *p* < 0.05; **, *p* < 0.01).

## Data Availability

All date and materials are available from the corresponding authors upon request.

## Abbreviations

TCGA: The Cancer Genome Atlas
HCC: Hepatocellular carcinoma
NF-YA: Nuclear transcription factor Y subunit alpha
qRT-PCR: Quantitative reverse transcription PCR
CDCA8: Cell division cycle associated 8
TMA: Tissue microarray
MAPK: Mitogen-activated protein kinase
CPC: Chromosomal passenger complex
TPM3: Tropomyosin 3
NECAP2: NECAP endocytosis associated 2
USP13: Ubiquitin specific peptidase 13
