# Supplementary figures and images for: CDCA8 induced by NF-YA promotes hepatocellular carcinoma progression by regulating the MEK/ERK pathway
Source: Exp Hematol Oncol. 2023 Jan 13;12:9. doi: 10.1186/s40164-022-00366-y (PMC9838039; doi:10.1186/s40164-022-00366-y)

a

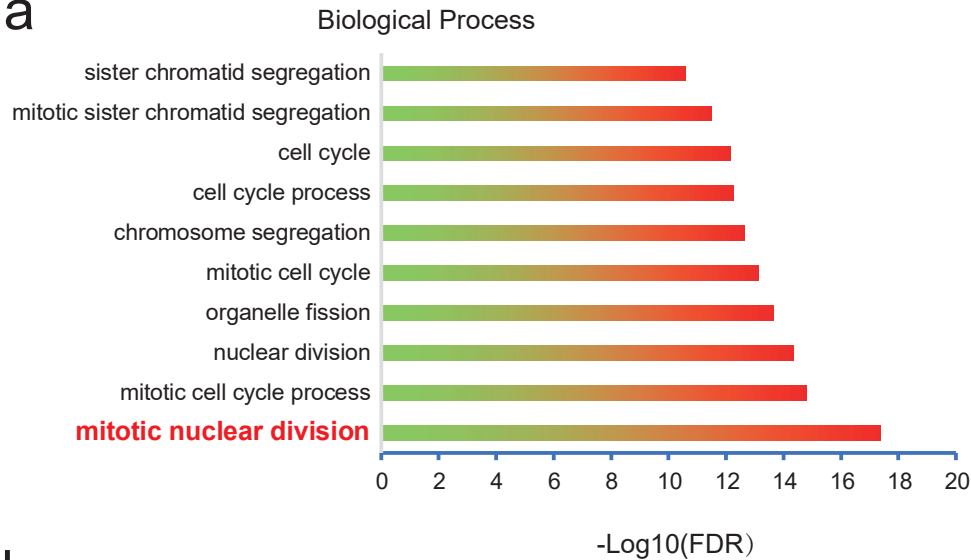

b

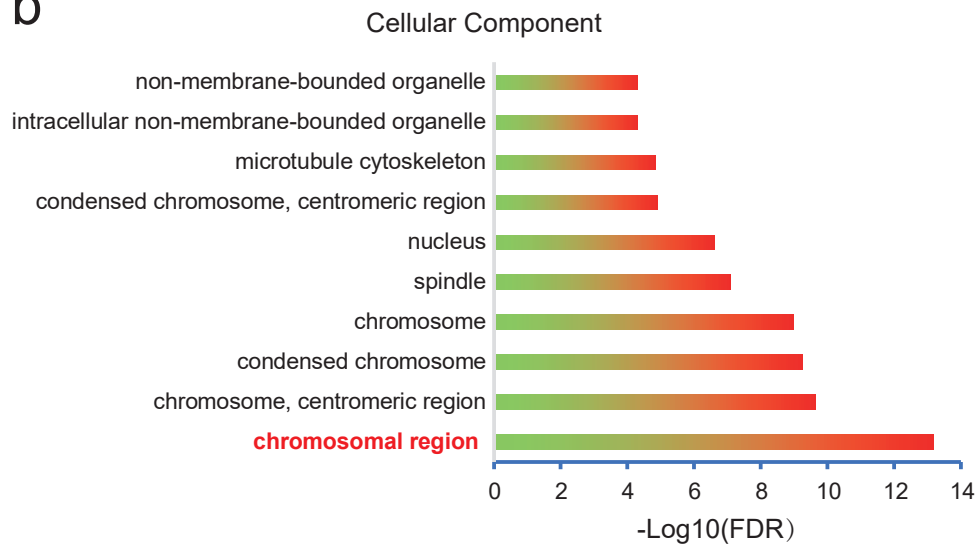

c

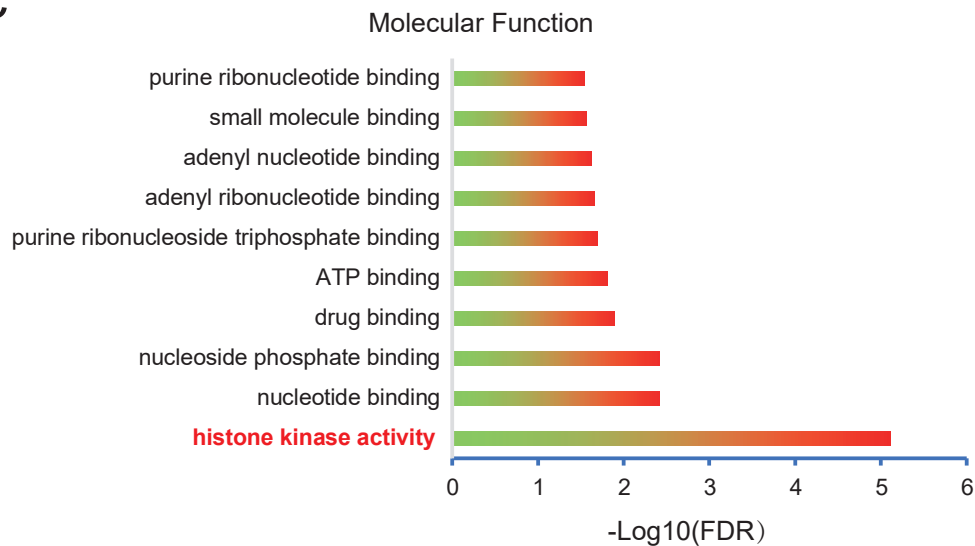

Supplement: Supplementary file 1 — Additional file 1: Figure S1. Gene ontology enrichment analysis of 36 genes. a Mitotic nuclear division was main annotation in the biological process category. b Chromosomal region was main annotation in the cellular component category. c Histone kinase activity was main annotation in the molecular function category. [file 40164_2022_366_MOESM1_ESM.pdf]

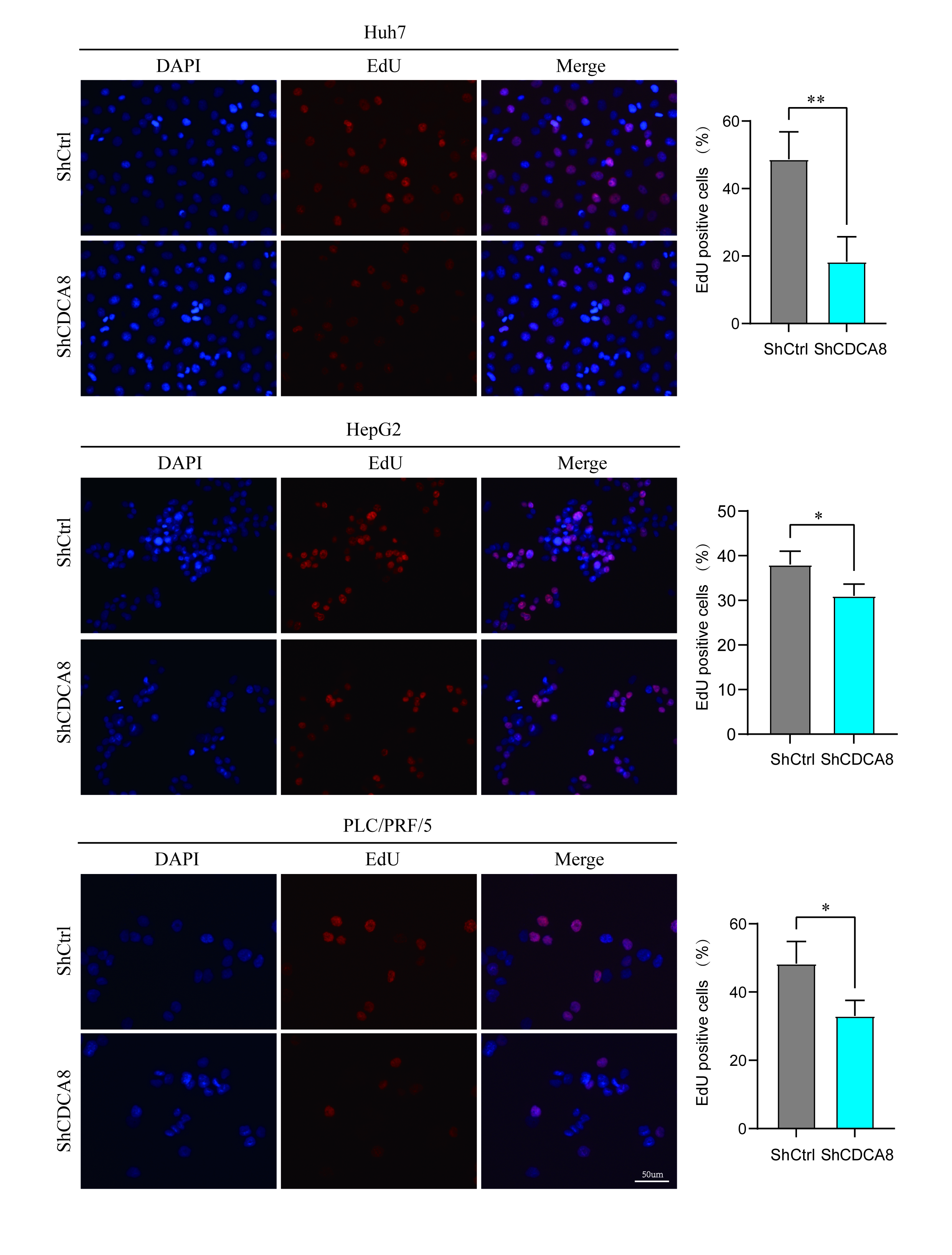

Supplement: Supplementary file 3 — Additional file 3: Figure S3. HepG2, PLC/PRF/5 and Huh7 cells were seeded onto coverslips and DNA synthesis was assessed via EdU immunofluorescence staining. (*, p < 0.05; **, p < 0.01). [file 40164_2022_366_MOESM3_ESM.tif]
