# Supplementary material for: CDCA8 induced by NF-YA promotes hepatocellular carcinoma progression by regulating the MEK/ERK pathway
Source: Exp Hematol Oncol. 2023 Jan 13;12:9. doi: 10.1186/s40164-022-00366-y (PMC9838039; doi:10.1186/s40164-022-00366-y)
Supplement: Supplementary file 2 — Additional file 2: Figure S2. The network of 26 genes. a The pathway underlying 36 genes. b MCODE analysis revealed that two core hub genes among 36 genes. [file 40164_2022_366_MOESM2_ESM.pdf]

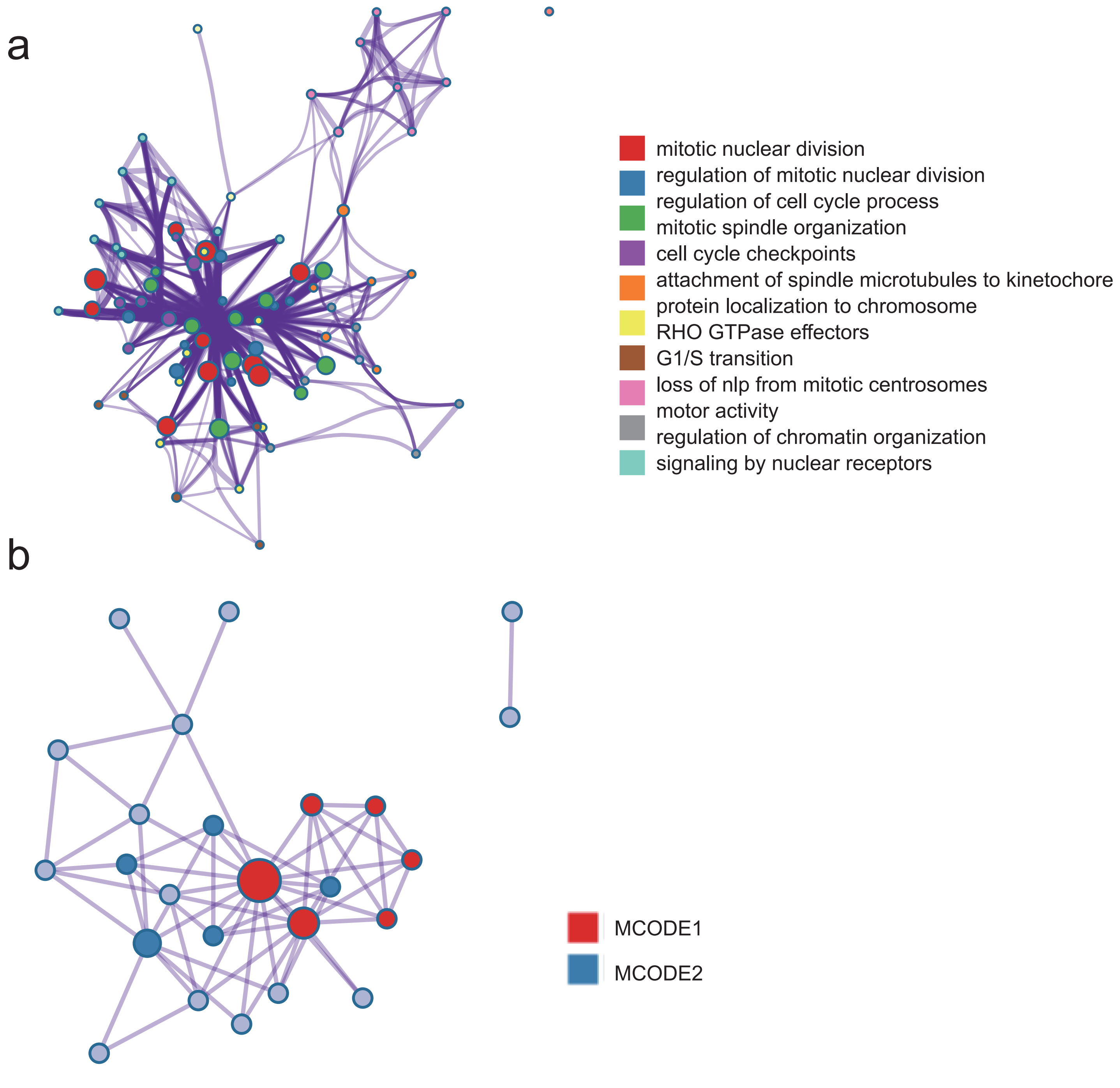
a
mitotic nuclear division
regulation of mitotic nuclear division
regulation of cell cycle process
mitotic spindle organization
cell cycle checkpoints
attachment of spindle microtubules to kinetochore
protein localization to chromosome
RHO GTPase effectors
G1/S transition
loss of nlp from mitotic centrosomes
motor activity
regulation of chromatin organization
signaling by nuclear receptors
b
MCODE1
MCODE2
